# Using Interpersonal Dimensions of Personality and Personality Pathology to Examine Momentary and Idiographic Patterns of Alliance Rupture

**DOI:** 10.3389/fpsyg.2021.711109

**Published:** 2021-08-16

**Authors:** Xiaochen Luo, Christopher J. Hopwood, Evan W. Good, Joshua E. Turchan, Katherine M. Thomas, Alytia A. Levendosky

**Affiliations:** ^1^Department of Counseling Psychology, Santa Clara University, Santa Clara, CA, United States; ^2^Department of Psychology, University of Zurich, Zurich, Switzerland; ^3^Department of Psychology, Michigan State University, East Lansing, MI, United States; ^4^Counseling & Psychiatric Services, Michigan State University, East Lansing, MI, United States; ^5^Center for Therapeutic Assessment, Austin, TX, United States

**Keywords:** Alternative Model of Personality Disorders (AMPD), interpersonal circumplex model, alliance rupture, momentary processes, idiographic analysis, psychotherapy process

## Abstract

The Alternative Model of Personality Disorders (AMPD) integrates several theoretical models of personality functioning, including interpersonal theory. The interpersonal circumplex dimensions of warmth and dominance can be conceptualized as traits similar to those in AMPD Criterion B, but interpersonal theory also offers dynamic hypotheses about how these variables that change from moment to moment, which help to operationalize some of the processes alluded to in AMPD Criterion A. In the psychotherapy literature, dynamic interpersonal behaviors are thought to be critical for identifying therapeutic alliance ruptures, yet few studies have examined moment-to-moment interpersonal behaviors that are associated with alliance ruptures at an idiographic level. The current study examined the concurrent and cross-lagged relationships between interpersonal behaviors and alliance ruptures within each session in the famous Gloria films (“Three Approaches to Psychotherapy”). Interpersonal behaviors (warmth and dominance) as well as alliance ruptures (i.e., withdrawal and confrontation) were calculated at half minute intervals for each dyad. We identified distinct interpersonal patterns associated with alliance ruptures for each session: Gloria (patient)’s warmth was positively related with withdrawal ruptures concurrently in the session with Carl Rogers; Gloria’s dominance and coldness were related with increased confrontation ruptures in the session with Fritz Perls concurrently, while her coldness was also predicted by confrontation ruptures at previous moments; lastly, both Gloria’s dominance and Albert Ellis’s submissiveness were positively related with withdrawal ruptures. These interpersonal patterns demonstrated the promise of using AMPD dimensions to conceptualize momentary interpersonal processes related to therapy ruptures, as well as the clinical importance of attuning to repetitive, dyad-specific interpersonal cues of ruptures within each session.

## Introduction

Conflicts are inevitable in close relationships, and therapeutic relationships are no exceptions. The term “alliance rupture” is used to refer to interpersonal conflicts, strains, or impasses that interfere with the therapeutic alliance between therapist and patient in psychotherapy (Safran and Muran, 2006). Alliance ruptures are prevalent in psychotherapy and can range from subtle, non-verbal disagreements to recurrent, intense conflicts (Coutinho et al., 2014). Alliance ruptures are associated with psychotherapy outcomes across a range of theoretical orientations (Safran, 1993; Muran et al., 2009). Unrepaired alliance ruptures can pose challenges for the therapeutic alliance and lead to premature terminations (Sharf et al., 2010; Eubanks et al., 2018). Repaired ruptures, on the other hand, can enhance the therapeutic alliance, and have been shown to relate to better outcomes compared to psychotherapy with unrepaired ruptures or without ruptures in a recent meta-analysis (Eubanks et al., 2018).

In this paper, we present a proof-of-concept design in which dimensions of personality disorder, as represented by the interpersonal circumplex model of personality, are conceived as dynamic factors that can vary from moment-to-moment within psychotherapy sessions and are linked to psychotherapy alliance ruptures. Using the well-known Gloria films, we demonstrate idiographic patterns linking variation in warmth and dominance with variation in confrontation and withdrawal ruptures in each of the three sessions. This work demonstrates that marrying evidence-based personality assessment with the dynamic relational processes that occur in psychotherapy has significant potential to augment research-practice integration, such that personality diagnostic variables can be directly informative regarding the specific processes such as alliance ruptures that are critical for effective psychotherapy. In particular, we argue in this paper that (a) interpersonal theory offers a model that can integrate the functional and structural dimensions of personality disorder as reflected in the DSM-5 Alternative Model for Personality Disorders (AMPD) within the interpersonal domain, (b) interpersonal dimensions and processes can be directly connected to clinically relevant variables such as alliance ruptures, and c) this has broad implications for researchers fine-tuning models such as the AMPD to better capture the structure and functioning in personality pathology.

### Identifying Therapeutic Alliance Ruptures

The observation that ruptures vary within psychotherapy relationships across time highlights the importance of identifying ruptures as they occur, so that they can be repaired effectively. The therapist’s awareness of how ruptures unfold in real time is thus a critical aspect of effective psychotherapy. Indeed, greater awareness of ruptures is related to better treatment outcomes (Chen et al., 2018). However, identifying ruptures can be challenging. Rupture expressions can be subtle (Muran and Eubanks, 2020). Patients may withhold dissatisfaction to please the therapist or avoid conflicts (Lambert, 2007). Therapists may be overwhelmed by their own anxiety in the moment and overlook the signals of ruptures. For all these reasons, ruptures may only be identified after they have escalated, making them more difficult to repair. Given the importance and complexity of identifying ruptures in real time, examining momentary signals of rupture development is critical for clinical practice.

Clinicians and supervisors often use two types of cues to identify ruptures: feelings and behaviors. As ruptures arise, a therapist’s own feelings or countertransference can send unique messages, prompting the therapist to attune to the challenges in the session. However, these transient, subjective feelings are difficult to assess in the moment for research; the typical way of asking a therapist to retrospectively report feelings after each session can only assess the average reaction toward the whole session but cannot capture changes in feelings as cues for ruptures within each session. Therefore, behavioral cues are critical for psychotherapy researchers to understand the momentary changes as ruptures unfold.

Among behavioral cues of ruptures, interpersonal behaviors have become the main focus in identifying ruptures given that a rupture is inherently a two-person phenomenon. Researchers have proposed a rupture-repair model, in which they differentiated ruptures into two types based on their interpersonal styles, namely withdrawal ruptures and confrontation ruptures (Eubanks et al., 2015). Withdrawal ruptures happen when either or both people “move away” from therapeutic interactions, with patient’s behavior markers such as avoiding directly talking about their difficulties, falling silent, responding minimally, changing topics, or being overly compliant. In contrast, confrontation ruptures happen when either party or both people “move against” each other, with patient’s behavior markers such as directly expressing blame, anger, resentment, or dissatisfaction toward the therapist or the treatment (Eubanks et al., 2019). These two subtypes have been shown to be associated with differentiated treatment strategies and consequences (Boritz et al., 2018). However, thus far connections have not been established between the withdrawal and confrontation ruptures and evidence-based dimensions of personality assessment.

### Interpersonal Theory and the Alternative Model of Personality Disorders (AMPD)

Interpersonal theory is an established model of personality, psychopathology, and psychotherapy with roots in the theoretical work of Sullivan (1953). A relatively unique feature of interpersonal theory is that it emphasizes both the structure of personality traits, as commonly instantiated in the interpersonal circumplex model (Leary, 1957; Locke, 2010), as well as dynamic processes and functions, such as complementarity that captures the dyadic interpersonal behavioral patterns (Carson, 1969). By integrating structure and function, interpersonal theory offers a model of personality functioning that can be used to both describe how people differ from another, and to articulate functional patterns that occur while people interact with one another (Wright et al., under review; Sadikaj et al., 2013; Dowgwillo et al., 2019; Hopwood et al., 2019; Ringwald et al., 2020).

The AMPD is also an integrative model of personality functioning that includes both structural and functional elements. AMPD Criterion B consists of a set of traits that are organized similarly to the five-factor model of personality, and which can be used to describe how people differ from one another, on average and across most situations. This is useful for determining the presence of trait levels suggestive of personality disorder, as well as for distinguishing different kinds of personality disorder from one another. AMPD Criterion A, in contrast, articulates developmental, functional, and dynamic processes that allude to how people adapt to their environments, particularly regarding self and interpersonal functioning. Criterion A includes elements such as the capacity for empathy, the ability to achieve one’s goals, the degree to which individuals can develop and maintain intimate relationships, and the extent to which people can sustain a coherent and realistic sense of self. These dynamics concepts are widely deemed to be clinically and developmentally important but are challenging to operationalize in practice.

Importantly, Mulay et al. (2018) recently reported that, of the major paradigms of personality assessment, interpersonal theory was relatively unique in sharing common ground with both the A and B criteria of the AMPD. Like Criterion B, the interpersonal circumplex model can be used to delineate how people differ from one another, on average, that speaks to the likelihood and type of personality disorder symptoms (Wilson et al., 2017). Specifically, the interpersonal circumplex dimensions of dominance/agency and warmth/communion can be recast as the Criterion B personality traits Detachment (i.e., low warmth or cold-submissiveness) and Antagonism (i.e., high dominance or cold-dominance) (Wright et al., 2012). Like Criterion A, interpersonal concepts like complementarity speak to the degree to which a person is able to function in specific environments in a manner that is either adaptive or adaptive. Of course, there are also differences between interpersonal theory and the AMPD. The IPC only contains two of the five Criterion B domains, and whereas Criterion A alludes to the outcomes of interpersonal functions, concepts like interpersonal complementarity operationalized them relatively more directly (Pincus and Roche, 2019). However, the IPC provides a model within which the general distinction between structure and function that underlies the AMPD can be examined. In this study, focus on how the kinds of adaptive personality functions alluded to by Criterion A can reveal important information about psychotherapy alliance ruptures.

### Models of Personality to Delineate Dynamic Patterns in Psychotherapy

The focus of interpersonal models and AMPD on functions offers a potential bridge between personality assessment and clinical practice. This is important because personality assessment has historically been conceptualized at a level that is too distal to meaningfully inform treatment processes such as alliance rupture patterns that are often considered critical by clinicians (Hopwood et al., 2015). Indeed, one longstanding controversy in personality diagnosis research is between the dimensional trait model of personality (the structure model; i.e., Criterion B) is focused on between-person differences that are relatively stable across situations, and the impairments that occur within specific contexts (the function model, i.e., Criterion A) but stand upon a smaller body of empirical evidence (Wright and Kaurin, 2020).

Several previous authors have suggested that the AMPD has considerable potential to augment clinical practice (Hopwood, 2018; Bach and Bernstein, 2019; Ruggero et al., 2019; Eubanks and Hunter, 2020; Hopwood et al., 2020a; Mullins-Sweatt et al., 2020). As described above, the AMPD is similar with interpersonal theory in combining an evidence-based model of personality structure with a model of adaptive functions that manifest in actual proximal situations. However, it remains unclear whether the AMPD as it currently exists is capable of distinguishing structure from function. Empirical studies have found that both Criterion A and Criterion B capture individual differences in personality structures and that there are significant overlaps between structures captured by the two criteria (Morey et al., 2020; Sleep et al., 2020). This can be attributed at least in part to the use of questionnaires in cross-sectional data, which likely increases overlap between Criterion A and B and also limits the degree to which the kinds of functions alluded to by Criterion A can be effectively operationalized.

Because of the current limitations in direct applications to capture dynamic therapeutic process in AMPD, we use interpersonal theory in this paper instead. Interpersonal theory offers a model of personality that (a) aligns closely with the AMPD model for both Criterion A and Criterion B conceptually and empirically (Wright et al., 2012; Dowgwillo et al., 2018; Pincus, 2018); (b) can do a better job in integrating and distinguishing the structure and the functioning of personality than the current AMPD model; and (c) can be applied to understand dynamic processes in clinical practice to illustrate the connection between the relatively abstract AMPD concepts with dynamic clinical phenomena (Thomas et al., 2014; Sadler et al., 2015).

### Mapping Dynamic Interpersonal Assessment With Momentary Alliance Ruptures in Psychotherapy

**FIGURE 1:**
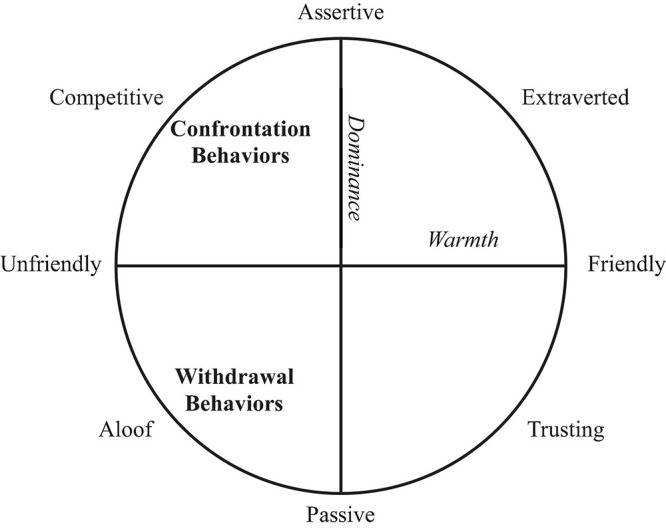
Interpersonal Circumplex at the behavioral level and the hypothesized relationships with confrontation and withdrawal rupture behaviors. Confrontation Ruptures are hypothesized to relate to cold-dominant behaviors, whereas withdrawal ruptures are hypothesized to relate to cold-submissive behaviors.

Alliance ruptures have been conceptualized as breakdowns between patients and therapist in negotiating their respective needs for “self-definition” and “relatedness” (Muran and Eubanks, 2020), which are inherently connected to the interpersonal dimensions of dominance and warmth, respectively (Wiggins, 1991). The style of withdrawal and confrontation ruptures may also be reconceptualized through the lens of interpersonal theory: confrontation ruptures likely manifest in cold-dominant interpersonal behaviors, whereas withdrawal ruptures likely manifest in cold-submissive behaviors (see the hypothesized relationships in Figure 1).

However, despite the direct theoretical connection and hypothesized relationships, the association between interpersonal behaviors and withdrawal and confrontation ruptures have never been explicitly examined at a moment-to-moment level. Examining the convergence and divergence of momentary assessments based on the rupture-repair model and the interpersonal theory is critical in at least four ways. First, evidence about the probability of different kinds of ruptures given different interpersonal styles can help clinicians understand whether and how rupture manifestations might be predicted using the terms of general interpersonal behaviors from the interpersonal theory. Given the wide use of interpersonal assessments across social and clinical situations and its connection with personality diagnoses and assessment (Dawood et al., 2018), connecting the rupture model that exclusively operationalizes behaviors during therapeutic impasses with the interpersonal model can help bridge research and practice based on alliance ruptures to the broader personality literature of understanding human relationships, conflicts, and personality pathology (Pincus, 2005).

Second, the rupture-repair model explicitly considers the underlying motivations for behavior in the conceptualization of ruptures on the underlying motivation (i.e., whether the patient is trying to move away). Motivation is also considered central in interpersonal theory (Horowitz, 2004; Wright et al., 2020). However, most momentary assessment tools for capturing interpersonal behaviors focus on behavior, somewhat independent of motivation (e.g., Sadler et al., 2009). For example, if someone actively initiated overly friendly behaviors, this may be viewed as withdrawal in the rupture-repair model if the intention is to avoid expressing authentic feelings or actual therapy work, whereas it may be assessed as warm and slightly dominant behaviors in the interpersonal theory framework, primarily based on the observable behaviors. Therefore, understanding how these two frameworks converge and diverge may help clarify the relationships between interpersonal motivation and behaviors during ruptures.

Third, although both frameworks hold a dyadic perspective, the assessment of dyadic behaviors differ: the rupture-repair model assesses overall ruptures at the dyadic level (Eubanks et al., 2015), whereas momentary assessment tools within the interpersonal model assess both people separately on the same interpersonal circumplex dimensions. This allows researchers to compare therapists’ and patients’ behaviors on the same scale and specify individuals’ contributions to dyadic relationships (Sadler et al., 2009). Therefore, assessing the relationships between alliance ruptures and interpersonal behaviors of warmth and dominance will inform how therapist’s and the patient’s behaviors may contribute together or separately to alliance ruptures.

Lastly, rupture models have thus far not been applied widely to examine moment-to-moment change, even though rupture processes can vary over the course of a session. Quantifying moment-to-moment changes in ruptures will allow for a more direct application of the framework to clinical practice in real time. In contrast, examination of within-session patterns of ruptures can inform us regarding how ruptures developed and decreased within each session and what interpersonal behaviors from either the patient or the therapist was predictors, signals, or consequences of ruptures (e.g., Altenstein et al., 2013; Thomas et al., 2014; Sadler et al., 2015; Li et al., 2020). If interpersonal behaviors at previous moments are predictors of certain ruptures later, clinical discussions may focus on understanding why these interpersonal behaviors represent recurrent triggers for therapeutic tension. If interpersonal behaviors are concurrent signals of ruptures, these signals may be used to help the therapist quickly identify the presence of ruptures. If certain interpersonal behaviors are consequences of previous ruptures, the therapist can be more aware of how ruptures pulled for certain interpersonal behaviors and how these behavioral consequences may reflect maladaptive interpersonal cycles (Hopwood, 2018). This information cannot be obtained by studying between-session patterns of ruptures, as is the norm in previous work on rupture processes.

### The Present Study

Given that no studies have examined momentary interpersonal processes in ruptures using a framework that connects with personality diagnostic variables and assessment, we selected the well-known Gloria videos (Shostrom, 1966) to demonstrate the approach, and to examine the within-session relationship between ruptures (i.e., withdrawal and confrontation) and interpersonal behaviors (i.e., warmth and dominance) of patients and therapists at a relatively momentary level (e.g., 30-s interval).

The advantage of examining the Gloria films is that it is one of the rare videos that demonstrate psychotherapy with the same patient and three different therapists (Barbosa et al., 2017); thus, it is possible to compare dyad-specific interpersonal patterns in ruptures with the same patient. Additionally, while the Gloria films have been one of the most studied psychotherapy demonstration videos (e.g., Kiesler and Goldston, 1988; Thomas et al., 2014), no studies have examined the momentary development of alliance ruptures and their interpersonal manifestations in these videos. The premise of this study is that doing so may provide a model for research that could ultimately inform practitioners regarding how the challenges in building therapeutic alliance arise and deescalate in concert with interpersonal dynamics. The Gloria videos also provide a rich depiction of the compelling ruptures, and a model of how different experts provoke and manage them in real time. Given that each session is quite different, these sessions allow us to test for idiographic patterns that may or may not generalize from one therapist to another.

In summary, the goal of this study is to illustrate how evidence-based personality assessments can be used to inform clinical meaningful phenomena of alliance ruptures, which we argue has implications for the AMPD model of personality functioning. We examined the unfolding of ruptures and their relationships with dyadic interpersonal behaviors on warmth and dominance momentarily for each session in the Gloria’s films. To facilitate the integration of quantitative examination with qualitative understanding, we also provided transcripts in selected sections to illustrate the identified associations between ruptures and interpersonal behaviors.

## Materials and Methods

### Session Materials

We used Shostrom’s (1966) “Three Approaches to Psychotherapy” films (also known as the Gloria films). In this set of the films, three therapists from three different approaches conducted one session each with the same patient named Gloria: Carl Rogers for Client-centered therapy, Frederick (Fritz) Perls for Gestalt therapy, and Albert Ellis for Rational-Emotive Behavior therapy. Client-centered therapy focuses on providing support, empathy, and trust in helping the patient to express their emotions and make their own decisions (Rogers, 1946, 1957). Gestalt therapy focuses on helping the patient take responsibility for their own experiences in life (Greenberg, 1997). Rational-emotive behavior therapy focuses on identification and disputation of the patient’s irrational beliefs (Ellis, 1986). The monologs of therapists discussing their approaches and reviewing the sessions were not used in the study; only the portion capturing interactions between Gloria and therapists was used.

The videos, data, training materials and reliability calculations for assessments, and example syntax for analysis are shared publicly here: https://osf.io/k6dh8/.

### Assessments

#### Alliance Ruptures

We used a modified version of Rupture-Resolution-Rating system to assess momentary fluctuations in ruptures (3RS; Eubanks et al., 2015, 2019). The 3RS was chosen in this study because it is one of the few validated systems to capture changes of alliance ruptures. The 3RS is an observer-rating system designed to assess withdrawal ruptures and confrontation ruptures over the course of a session and can be used to generate time series of ruptures. Withdrawal ruptures and confrontation ruptures are evaluated in separate categories in the 3RS. Some of the example markers for withdrawal ruptures include denial, giving minimal response, shifting the topic, and being deferential in patient’s behaviors. Some of the example markers for confrontation ruptures include complaining about the therapist, complaining about the progress of therapy, rejecting intervention, and pressuring the therapist. The rupture markers were assessed as being present (rated as 1) or absent (rated as 0) within each segment. Coders also assess the significance of ruptures on the alliance at the end of each session on a 1-to-5 Likert scale, with 1 indicating no rupture and 5 indicating very significant rupture on the alliance.

The 3RS was modified for the current study in two ways. First, the segment for assessing ruptures was modified from 5-min intervals to 30-s intervals to obtain a larger number of data points for each session while also detecting meaningful ruptures (Sadler et al., 2009). Second, raters were asked to assess the significance of ruptures for each 30s window instead of the entire session, with 1 indicating no or little rupture that has no impacts on the alliance and 5 indicating very intense rupture that has significant impact on the alliance. These significance ratings were given based on the rater’s global sense of the degree of rupture in the segment in addition to the mere occurrence of one or more ruptures during that segment, although raters also used rupture markers in each segment to inform their overall rating. If a rupture was present across multiple segments, it is reflected in the ratings of all of the relevant segments. The significance scores were assessed separately for withdrawal ruptures and confrontation ruptures and were used in the final analyses.

Ruptures were rated by four trained coders including two advanced Ph.D. students in clinical psychology and two licensed clinical psychologists, given that the 3RS requires understanding of the therapeutic alliance. All 3RS coders were blind to the interpersonal coding of the Gloria films. They were trained for at least 8 h for 3RS coding. Two coders attended the in-person training workshop for 3RS coding from one of the developers of 3RS, Dr. Eubanks. Prior to coding the study videos, all of the coders coded for at least 16-h of actual psychotherapy sessions using the modified 3RS protocol and had weekly training meetings to discuss coding discrepancies, until their Intra-Class Correlation coefficients (ICC) on practice videos reached a benchmark of 0.40. The inter-rater reliability of ruptures was assessed by calculating the two-way mixed, average-measure, absolute agreement intra-class correlation coefficients (ICCs) over the rupture time series for each entire session (Hallgren, 2012). Given that no prior studies have calculated ICCs for 3RS based on scores assessed every half minute, a suggestive benchmark of 0.40 was used as a cut-off score for evaluating fair reliability (Cicchetti, 1994). In the current study, ICCs for withdrawal ruptures are 0.43, 0.64, and 0.40 for Rogers, Perls, and Ellis, respectively. The ICCs for confrontation ruptures are 0.73, 0.79, and 0.82, respectively. ICCs are higher for confrontation ruptures than withdrawal ruptures, indicating challenges with coding more subtle expressions of ruptures.

#### Interpersonal Behaviors

We used the momentary interpersonal behaviors data presented in Thomas et al. (2014) that were measured at the half-second interval and aggregated the data on a 30-s interval in the current study. Briefly, we used the Continuous Assessment of Interpersonal Dynamics (CAID; Sadler et al., 2009) to assess interpersonal behaviors of warmth and dominance for both patient and therapist. CAID was chosen because this method can capture relatively continuous streams of interpersonal behaviors in both parties as a dyadic interaction unfolds. The details of CAID are extensively described in previous studies (e.g., Sadler et al., 2009; Lizdek et al., 2012; Thomas et al., 2014; Hopwood et al., 2020b; Fox et al., 2021). CAID provides observer-rated time series of the interpersonal circumplex dimensions of warmth and dominance for dyadic interactions (Girard and Wright, 2018). The warmth dimension describes behaviors ranging from being warm, friendly, and close, to being cold, unfriendly, and distant. The dominance dimension describes behaviors ranging from being dominant and taking control, to being submissive and giving control. A variety of interpersonal behaviors can be described as blends of the two dimensions, such as being withdrawn (cold-submissive), cooperative (warm-submissive), critical (cold-dominant), or gregarious (warm-dominant) (Leary, 1957; Wiggins, 1979). Further, the extremity of behaviors along these dimensions can be rated based on their proximity to the center (neutral). For instance, a slightly critical comment would be rated as a little cold and a little dominant, whereas an extremely critical comment would be rated as highly cold and highly dominant.

Trained coders use a computer joystick device to assess dominance and warmth for each person, one at a time, as they watch a video-taped interaction. The computer monitor displays the target video on one side as well as a Cartesian plane depicting the interpersonal circumplex dimensions of dominance and warmth on the other side. A dot moves within the Cartesian plane in accordance with joystick movements, allowing coders to view the placement of their ratings as they watch videos. By using the joystick to move this dot on this circumplex, coders can indicate shifts in interpersonal behaviors on both or either of the dimensions. CAID data are scaled from −1,000 to 1,000 on both dimensions, with 1,000 on the y-axis representing extreme dominance and 1,000 on the *x*-axis representing extreme warmth. The dominance and warmth scores are captured by the computer program called Dual Axis Rating and Media Annotation (DARMA; Girard and Wright, 2018) every half second.

The procedure of training coders, cross-examination of coding-recording reliabilities, and cleaning the data was described elsewhere (Thomas et al., 2014). In brief, six trained coders contributed to the time series of dominance and warmth for Gloria and each therapist in each session. To calculate reliability, the researchers estimated the true score variance as the mean of the cross covariances of the individual raters’ times series and estimated the total variance as the variance of the aggregated time series. The reliability overall was 0.80 for dominance and 0.66 for warmth across sessions using a standard approach described by Sadler et al. (2009; Thomas et al., 2014). To match the interval of 30 s for rupture variables, the original data for interpersonal behaviors in the Thomas et al.’s study (2014) were averaged for every 30 s in the current study and the aggregated time series were used in the further analyses.

### Data Analyses

We used Dynamic Structural Equation Modeling (DSEM) to model the multivariate within-session relationships between dyadic interpersonal behaviors and alliance ruptures (Asparouhov et al., 2018). DSEM is a method developed to model individual time series, extract individualized structures, and to quantify associations between multiple variables over time within a structural equation modeling (SEM) framework. Mplus 8.2 was used to conduct all the Dynamic Structural Equation Models (DSEM). Bayes estimation was used to handle the non-normal distribution of rupture variables (Wang et al., 2015; Yang et al., 2015; Muthén and Muthén, 1998–2017). For small datasets with non-normal distributions, Bayesian estimation outperforms maximum likelihood estimation in terms of both bias and precision for small datasets with non-normal data (Ghosh et al., 2006).

The DSEM models in the current study included six variables for each session: patient’s warmth, patient’s dominance, therapist’s warmth, therapist’s dominance, withdrawal rupture, and confrontational rupture. The model was specified to include (1) an autoregression effect for each variable, in which one variable at time t predicts its state at time *t* + 1; and (2) the concurrent associations among interpersonal variables and among rupture variables. The concurrent associations (covariance) between interpersonal variables (patient’s warmth, patient’s dominance, therapist’s warmth, therapist’s dominance) and rupture variables (withdrawal rupture and confrontational rupture) were estimated by default.

Once we identified significant concurrent correlations between interpersonal variables and rupture variables, we further used cross-lagged models using DSEM to determine the nature and the directionality of the covariance between the interpersonal behavior variables and rupture variables. The cross-lagged regressions from interpersonal behaviors at time *t* to ruptures at time *t* + 1 and from ruptures at time *t* to interpersonal behaviors at time *t* + 1 was added to the original models to examine potential directional, lagged associations. Regression coefficients and p-values were obtained using the Bayes estimation in all DSEM models.

After identifying significant associations, we selected sections of transcripts to illustrate how the associations manifest in interactions. We supplemented these transcripts with graphical depiction of the raw time series to provide a more nuanced depiction of how these associations played out during the session.

## Results

**FIGURE 2:**
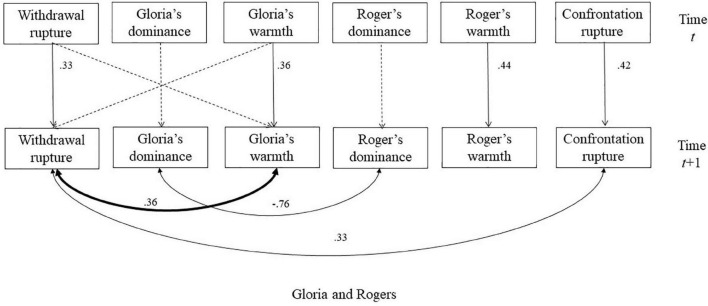
The selected model for the session with Rogers. The dash lines indicated non-significant autoregressive or cross-lagged regressive pathways. Significant parameters were noted next to solid lines. The double-arrowed lines indicated covariance and the single-arrowed lines indicated autoregressive or cross-lagged regressive pathways. The significant relationships between interpersonal behaviors and ruptures were bolded.

**FIGURE 3:**
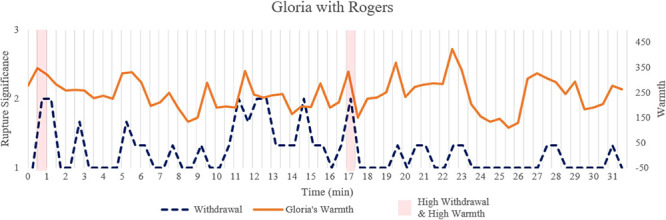
Time series and selected segments for transcripts in the session with Rogers. This figure presents the time series data for withdrawal rupture and Gloria’s warmth to illustrate the significant association. The areas shaded with light red corresponded to the transcripts quoted in the text to illustrate moments with increased withdrawal and increased warmth.

**FIGURE 4:**
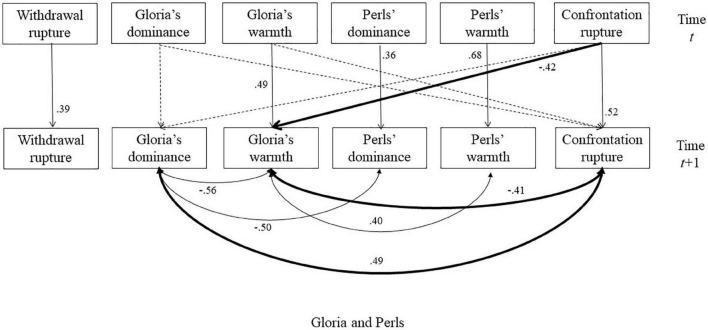
The selected model for the session with Perls. The dash lines indicated non-significant autoregressive or cross-lagged regressive pathways. Significant parameters were noted next to solid lines. The double-arrowed lines indicated covariance and the single-arrowed lines indicated autoregressive or cross-lagged regressive pathways. The significant relationships between interpersonal behaviors and ruptures were bolded.

**FIGURE 5:**
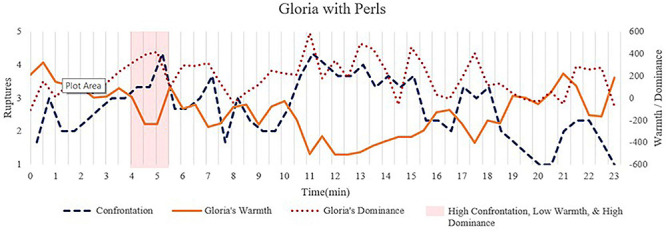
Time series of confrontation ruptures and Gloria’s interpersonal behaviors to illustrate their significant associations. The segments with light red corresponded to the transcripts quoted in the text to illustrate moments with increased dominance in Gloria, increased confrontation rupture, and decreased warmth.

**FIGURE 6:**
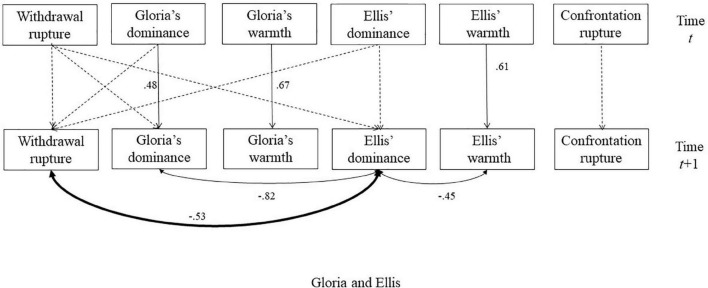
The selected model for the session with Ellis. The dash lines indicated non-significant autoregressive or cross-lagged regressive pathways. Significant parameters were noted next to solid lines. The double-arrowed lines indicated covariance and the single-arrowed lines indicated autoregressive or cross-lagged regressive pathways. The significant relationships between interpersonal behaviors and ruptures were bolded. The association between Gloria’s dominance and withdrawal ruptures became non-significant (*p* = 0.047, 95% CI is –0.06 to 0.69) after adding the cross-lagged associations.

**FIGURE 7:**
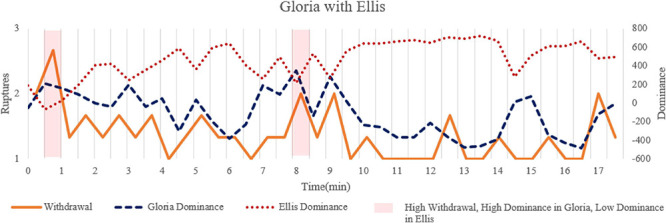
Time series of withdrawal ruptures and dominance in Gloria and Ellis to illustrate the significant associations. The segments with light red corresponded to the transcripts quoted in the text to illustrate moments with increased dominance in Gloria, increased withdrawal rupture, and decreased dominance in Ellis.

The descriptive statistics of interpersonal and rupture variables for each session were presented in Table 1. The parameters for each DSEM model for each session is presented in Tables 2–4. To facilitate understanding of the results, we describe each of the sessions separately at the dynamic, within-session level (Figures 2–7).

**TABLE 1:** Descriptives of ruptures and interpersonal behaviors.

	Dominance*		Warmth*		**Withdrawal ruptures**			**Confrontation ruptures**		
	***M***	***SD***	***M***	***SD***	***M***	***SD***	**% of segments with withdrawal markers**	***M***	***SD***	**% of segments with confrontation markers**
Carl Rogers	−99.01	89.91	249.75	39.04	1.43	0.51	14.1%	1.31	0.55	25.0%
Gloria (with Rogers)	123.03	244.38	243.45	80.05						
Fritz Perls	295.84	169.94	−11.23	113.56	1.60	0.67	23.4%	2.70	1.07	78.7%
Gloria (with Perls)	180.78	216.65	−127.56	232.72						
Albert Ellis	471.48	259.56	110.28	58.48	1.32	0.52	13.9%	1.34	0.59	27.8%
Gloria (with Ellis)	−116.17	283.59	105.36	108.5						

*The descriptives of dominance and warmth were reported in Thomas et al. (2014).

**TABLE 2:** Standardized parameter estimations for the final DSEM model for the session with Rogers.

**Parameter types**	**Variables**	**Estimate**	**Posterior *S.D*.**	***P*-value**	**95% CI**
Autoregressive parameters	**Gloria’s warmth**	**0.36**	**0.14**	**0.008**	**0.06 -0.64**
	Gloria’s dominance	0.11	0.11	0.18	−0.11 −0.32
	**Roger’s warmth**	**0.44**	**0.13**	**0.001**	**0.19**−**0.70**
	Roger’s dominance	0.03	0.12	0.42	−0.21 −0.25
	**Withdrawal rupture**	**0.33**	**0.14**	**0.01**	**0.04**−**0.59**
	**Confrontation rupture**	**0.42**	**0.13**	**0.001**	**0.17**−**0.69**
Within-person interpersonal correlations	Gloria’s warmth and Gloria’s dominance	0.16	0.14	0.14	−0.12 −0.42
	Roger’s warmth and Roger’s dominance	0.13	0.14	0.19	−0.17 −0.39
Between-person interpersonal correlations	Gloria’s warmth and Roger’s warmth	0.28	0.13	0.03	−0.01 −0.52
	**Gloria’s dominance and Roger’s dominance**	−**0.76**	**0.06**	**0.000**	−**0.86**−**0.62**
	Gloria’s warmth and Roger’s dominance	−0.15	0.14	0.16	−0.41 −0.14
	Gloria’s dominance and Roger’s warmth	−0.04	0.14	0.39	−0.31 −0.25
Correlations between withdrawal ruptures and interpersonal variables	Withdrawal and Gloria’s dominance	=0.14	0.14	0.18	−0.40 −0.15
	**Withdrawal and Gloria’s warmth**	**0.36**	**0.13**	**0.005**	**0.09**−**0.57**
	Withdrawal and Roger’s dominance	0.03	0.14	0.42	−0.24 −0.31
	Withdrawal and Roger’s warmth	0.09	0.14	0.27	−0.19 −0.35
Correlations between confrontation ruptures and interpersonal variables	Confrontation and Gloria’s dominance	−0.06	0.15	0.35	−0.34 −0.23
	Confrontation and Gloria’s warmth	−0.06	0.15	0.35	−0.34 −0.23
	Confrontation and Roger’s dominance	−0.20	0.14	0.08	−0.45 −0.07
	Confrontation and Roger’s warmth	−0.06	0.15	0.35	−0.34 −0.23
Rupture correlations	**Withdrawal and confrontation**	**0.33**	**0.14**	**0.02**	**0.03**−**0.55**
Cross-lagged effects*	Withdrawal at *t* → Gloria’s warmth at *t* + 1	0.05	0.13	0.35	−0.20 −0.33
	Gloria’s warmth at *t* → Withdrawal at *t* + 1	−0.03	0.14	0.40	−0.30 −0.24

*Bolded items indicated significance of *p* < 0.05. ^∗^*t* = 30 s in the cross-lagged modeling.*

**TABLE 3:** Standardized parameter estimations for the final DSEM model for the session with Perls.

**Parameter types**	**Variables**	**Estimate**	**Posterior *S.D*.**	***P*-value**	**95% CI**
Autoregressive parameters	**Gloria’s warmth**	**0.49**	**0.11**	**0.000**	**0.28 −0.70**
	Gloria’s dominance	0.22	0.16	0.09	**−**0.1 **−**0.52
	**Perls’ warmth**	**0.68**	**0.11**	**0.000**	**0.46 −0.88**
	**Perls’ dominance**	**0.36**	**0.13**	**0.004**	**0.11 −0.60**
	**Withdrawal rupture**	**0.39**	**0.17**	**0.01**	**0.06 −0.72**
	**Confrontation rupture**	**0.52**	**0.19**	**0.005**	**0.13 −0.88**
Within-person interpersonal correlations	**Gloria’s warmth and Gloria’s dominance**	**−0.56**	**0.13**	**0.000**	**−0.75 – −0.25**
	Perls’ warmth and Perls’ dominance	**−**0.30	0.16	0.04	**−**0.57 **−**0.03
Between-person interpersonal correlations	**Gloria’s warmth and Perls’ warmth**	**0.40**	**0.15**	**0.01**	**0.08 −0.64**
	**Gloria’s dominance and Perls’ dominance**	**−0.50**	**0.14**	**0.003**	**−0.72 – −0.19**
	Gloria’s warmth and Perls’ dominance	**−**0.03	0.17	0.44	**−**0.36 **−**0.30
	Gloria’s dominance and Perls’ warmth	**−**0.17	0.17	0.18	**−**0.48 **−**0.17
Correlations between withdrawal ruptures and interpersonal variables	Withdrawal and Gloria’s dominance				
	Withdrawal and Gloria’s warmth	0.12	0.18	0.25	**−**0.21 **−**0.47
	Withdrawal and Perls’ dominance	0.08	0.17	0.32	**−**0.27 **−**0.38
	Withdrawal and Perls’ warmth	0.03	0.17	0.43	**−**0.31 **−**0.34
Correlations between confrontation ruptures and interpersonal variables	**Confrontation and Gloria’s dominance**	**0.49**	**0.14**	**0.002**	**0.18 −0.72**
	**Confrontation and Gloria’s warmth**	**−0.41**	**0.15**	**0.01**	**−0.65 – −0.08**
	Confrontation and Perls’ dominance	0.05	0.18	0.39	**−**0.29 **−**0.38
	Confrontation and Perls’ warmth	**−**0.29	0.16	0.05	**−**0.57 **−**0.06
Rupture correlations	Withdrawal and confrontation	**−**0.15	0.17	0.19	**−**0.47 **−**0.19
Cross-lagged effects between ruptures and interpersonal behaviors*	**Confrontation at *t* → Gloria’s warmth at *t* + 1**	**−0.42**	**0.12**	**0.002**	**−0.63 – −0.17**
	Gloria’s warmth at *t***→** confrontation at *t* + 1	**−**0.26	0.16	0.06	**−**0.58 **−**0.07
	Confrontation at *t***→** Gloria’s dominance at *t* + 1	0.30	0.21	0.06	**−**0.07 **−**0.75
	Gloria’s dominance at *t* **→** confrontation at *t* + 1	**−**0.06	0.15	0.35	**−**0.33 **−**0.26

*Bolded items indicated significance of *p* < 0.05. ^∗^*t* = 30 s in the cross-lagged modeling.*

**TABLE 4:** Standardized parameter estimations for the final DSEM model for the session with Ellis.

**Parameter types**	**Variables**	**Estimate**	**Posterior *S.D*.**	***P*-value**	**95% CI**
Autoregressive parameters	**Gloria’s warmth**	**0.67**	**0.19**	**0.004**	**0.25 −0.97**
	**Gloria’s dominance**	**0.48**	**0.16**	**0.004**	**0.14 −0.78**
	**Ellis’ warmth**	**0.61**	**0.15**	**0.001**	**0.31 −0.89**
	Ellis’ dominance	0.26	0.16	0.05	**−**0.04 **−**0.55
	Withdrawal rupture	0.12	0.25	0.30	**−**0.38 **−**0.60
	Confrontation rupture	0.04	0.24	0.42	**−**0.53 **−**0.50
Within-person interpersonal correlations	Gloria’s warmth and Gloria’s dominance	**−**0.03	0.21	0.46	**−**0.42 **−**0.41
	**Ellis’ warmth and Ellis’ dominance**	**−0.45**	**0.18**	**0.01**	**−0.72 – −0.04**
Between-person interpersonal correlations	Gloria’s warmth and Ellis’ warmth	0.29	0.22	0.12	**−**0.20 **−**0.66
	**Gloria’s dominance and Ellis’ dominance**	**−0.82**	**0.08**	**0.000**	**−0.92 – −0.62**
	Gloria’s warmth and Ellis’ dominance	**−**0.12	0.21	0.30	**−**0.52 **−**0.30
	Gloria’s dominance and Ellis’ warmth	0.32	0.20	0.08	**−**0.16 **−**0.63
Correlations between withdrawal ruptures and interpersonal variables	Withdrawal and Gloria’s dominance	0.38	0.20	0.05	**−**0.06 **−**0.69
	Withdrawal and Gloria’s warmth	0.09	0.22	0.34	**−**0.34 **−**0.50
	**Withdrawal and Ellis’ dominance**	**−0.53**	**0.18**	**0.009**	**−0.78 – −0.09**
	Withdrawal and Ellis’ warmth	0.08	0.23	0.36	**−**0.41 **−**0.50
Correlations between confrontation ruptures and interpersonal variables	Confrontation and Gloria’s dominance	0.00	0.24	0.49	**−**0.45 **−**0.47
	Confrontation and Gloria’s warmth	**−**0.22	0.20	0.14	**−**0.56 **−**0.19
	Confrontation and Ellis’ dominance	0.06	0.22	0.39	**−**0.39 **−**0.49
	Confrontation and Ellis’ warmth	**−**0.31	0.19	0.06	**−**0.64 **−**0.11
Rupture correlations	Withdrawal and Confrontation	0.10	0.22	0.32	**−**0.36 **−**0.49
Cross-lagged effects*	Withdrawal at *t* → Ellis’ dominance at *t* + 1	**−**0.08	0.22	0.35	**−**0.51 **−**0.32
	Ellis’ dominance at *t* → withdrawal at *t* + 1	0.19	0.30	0.27	**−**0.42 **−**0.72

*Bolded items indicated significance of *p* < 0.05. ^∗^*t* = 30 s in the cross-lagged modeling.*

### Session With Rogers

Figure 2 illustrates significant findings in the cross-lagged model for the session with Rogers. There were significant positive autoregressions for Gloria’s warmth, Rogers’ warmth, and both rupture variables, which indicated that these variables at one moment were positively influenced by their own values 30 s ago. We found significant negative associations between Gloria’s and Roger’s dominance, which indicated dominance complementarity (Carson, 1969), such that when one person becomes more dominant, the other person becomes more submissive in the dyad. Withdrawal ruptures were positively related with confrontation ruptures, suggesting that withdrawal and confrontation ruptures tended to increase or decrease together within the session (Table 2).

Most pertinent for the goals of this study, we found a significant correlation between Gloria’s warmth and withdrawal ruptures, indicating that that withdrawal ruptures increased/decreased when Gloria became warmer/colder. We explored this correlation by including cross-lagged regressive pathways to examine whether Gloria’s warmth at a previous moment led to increases in withdrawal ruptures 30 s later and vice versa. We did not find any significant cross-lagged associations, indicating that Gloria’s increased warmth only co-occurred with increased ruptures without contributing to lagged changes in ruptures. This suggests that her warm behavior could be seen as an interpersonal signal, rather than an interpersonal predictor or consequence of withdrawal ruptures in this session.

To illustrate the associations between warmth and withdrawal ruptures, we highlighted two segments with high levels of withdrawal ruptures and high levels of Gloria’s warmth (segments of 0.5′–1′, 17′–17.5′; highlighted in light red in Figure 3). These transcripts revealed that Gloria’s affect mismatched the content of her spoken words in these segments, in that she frequently smiled when talking about her anxiety and hesitation:

Segment 1: (0.5′–1′ [light red in Figure 3])

*Gloria: Well I’m… (smiling) … right now (smiling) I feel nervous, but I feel more comfortable the way you are talking in a low voice. I don’t feel like you will be so harsh on me… But I …*

*Rogers: I hear the tremor in your voice…*

*Segment 2:* (17′–17.5′ [light red in Figure 3])

*Rogers: What I mean is you have been sitting here just telling me what you would like to do with Pam.*

*Gloria: I would, but I don’t want to quite take the risk, (smiling) unless an authority tells me that.*

This association indicated that warm behaviors may not necessarily indicate a strong alliance, but rather represented a way for Gloria to express discomfort, anxiety, or distance, especially in this session with Rogers. This disconnect between affect and verbal behavior may reflect a level of distrust in the patient and discomfort with the self that could interfere with psychotherapy and interpersonal functioning, and thus may be considered an important issue to address in psychotherapy.

### Session With Perls

Figure 4 illustrates the significant associations for the session between Gloria and Perls. We observed significant positive autoregressions for Gloria’s and Perls’ warmth and dominance as well as both rupture variables, which indicated that these variables at any given point in time were positively influenced by their own values 30 s previously (Table 3). The results also indicated significant negative associations between Gloria’s and Perls’ dominance and significant positive associations between their warmth, suggesting complementary patterns around warmth and dominance for the dyad in that when one person becomes warmer, the other person becomes warmer and when one person becomes more dominant, the other person becomes more submissive. Gloria’s warmth and dominance were negatively associated with each other, indicating that when she became more dominant, she tended to become colder too (see Fournier et al., 2008, for a discussion of this kind of pattern).

Importantly, Gloria’s dominance was positively associated with confrontation ruptures, indicating that for segments with more confrontation ruptures, Gloria was more dominant compared with her behaviors in other segments with fewer confrontation ruptures. Furthermore, Gloria’s warmth was negatively related with confrontation ruptures, meaning that Gloria tended to become colder during moments with more confrontation ruptures.

To further explore these correlations between confrontation and Gloria’s behaviors, we added the cross-lagged regressive pathways to examine whether Gloria’s dominance and warmth at a previous moment contributed to confrontation ruptures 30 s later, and/or if confrontation ruptures at one moment contributed to changes in dominance or warmth 30 s later. The results indicated that confrontation ruptures at one moment were associated with decreases in Gloria’s warmth 30 s later. We did not identify any significant cross-lagged associations between dominance and confrontation ruptures. Taken together, these results suggested that increases in confrontation ruptures manifest in concurrent increases in Gloria’s dominance and decreases in Gloria’s warmth, as well as contributing to decreases in Gloria’s warmth 30 s later. This suggests that, in the session with Perls, Gloria’s changes in dominance and warmth were concurrent signals of confrontation ruptures as well as consequences of confrontation ruptures.

We selected an exemplar transcript (4′–5.5′, light red in Figure 5) that contained one of the highest levels of confrontation ruptures early in the session to illustrate the identified associations. Gloria was provoked by Perls’ comments that she is “phony,” after which she became colder and more dominant:

*Perls: You are bluffing. You are a phony.*

*Gloria: Do you believe… do you mean that seriously?*

*Perls: Yeah… you’re laughing, giggling… that’s phony. You put on a performance for me.*

*Gloria: Oh, I, I resent that very much.*

*Perls: Can you explain it?*

*Gloria: Yes sir. I most certainly am not being a phony. I will admit this – it’s hard for me to show my embarrassment, and I hate to be embarrassed. But boy, I resent you calling me a phony. Just because I smile when I’m embarrassed or put in a corner, it doesn’t mean that I’m being a phony.*

*Perls: Wonderful. Thank you. (moves his hand as an offer to shake hands that Gloria rejects). You didn’t smile for the last minute.*

*Gloria: Well I’m mad at you! (smiling) I.*

*Perls: (interrupting) That’s, that’s right. You didn’t have to cover up your anger with your smile. At that moment, that moment, you are not a phony.*

*Gloria: Well at that minute I was mad though. I wasn’t embarrassed.*

*Perls: When you are mad you are not a phony.*

*Gloria: (waving her hand) I still resent that. I’m not a phony when I’m nervous. (beating the couch)*

*Perls: Again!*

*Gloria: I… (laughing, beating the coach again), I, want to get mad at you! I’m, I… You know what I…*

*Perls: (rudely interrupting) I, I, I!*

*Gloria: …want to do? I want you at my level, so I can pick on you, just as much as you are picking on me.*

*Perls: Ok, pick on me.*

*Gloria: (silence, smiling) I have to wait till you say something that I can pick on (moving her hands outwardly).*

In this exchange, Perls provoked Gloria by calling her “a phony.” Gloria saw this as disrespectful (“you are picking on me”) and became more dominant and colder as she confronted him. She directly expressed her resentment toward him and indicated that she was “mad.” Such a transition of Gloria to being colder and more dominant was highly encouraged by Perls (“Wonderful, thank you.” “at that moment you are not a phony,” “ok pick on me”) and the expression of coldness continued after his encouragement.

### Session With Ellis

Figure 6 illustrates the associations between ruptures and interpersonal behaviors for the session between Gloria and Ellis. The model identified significant positive autoregressions for Gloria’s and Ellis’ warmth and dominance, which indicated that these interpersonal variables at one segment were positively influenced by their own values at the previous segment. Consistent with findings in the other sessions, the results also indicated significant negative associations between Gloria’s and Ellis’ dominance, indicating complementarity in dominance.

Importantly, withdrawal ruptures were positively related to Gloria’s dominance and negatively related to Ellis’ dominance. These patterns indicated that increases in Gloria’s dominance and Ellis’ submissiveness, and increases in withdrawal ruptures co-occur in this session. Cross-lagged regressive pathways were not significant (Table 4). This pattern suggests that withdrawal ruptures manifested concurrently in the changes of dominance for both Gloria and Ellis. Interestingly, the association between Gloria’s dominance and withdrawal ruptures changed from being significant to marginally significant, indicating that the associations between dominance and withdrawal ruptures were perhaps stronger for Ellis than for Gloria.

We selected two segments with significant withdrawal ruptures to illustrate the associations (see Figure 7 for time series data):

Segment 1: (0.5′–1′ [light red in Figure 7])

*Gloria: I don’t know if I’m doing the wrong thing but I’m going to refer to your book anyway (smiling) because… this is what I’m impressed with, your book about ‘the intelligent woman’s guide to man-hunting.’*

*Ellis: Yeah.*

*Gloria: I try to follow it (laughing), and I’m believing in it. This is why it’s so fun reading your book because I’m not much of a reader, but I sort of believe the same way you do. But then I’ve got a problem with this area or theme, men that I do… men that I’m attracted to, or the type of men that I’d like to become closely involved with, I can’t seem to meet or I get too shy or something. I don’t….it just doesn’t click.*

Segment 2: (8′–8.5′ [light red in Figure 7])

*Gloria: I want a step toward moving forward.*

*Ellis: (interrupting her) What’s stopping you?*

*Gloria: I don’t know. I thought… What I was hoping is, whatever it is in me, why I don’t seem to be attracting this kind of man, why I seem to be more defensive why I seem more afraid, you could help me with what it is that I’m afraid of so I won’t do it so much.*

*Ellis: Well, my hypothesis so far is that what you’re afraid of is not just failing with this individual man, which is really the only thing at issue when you go out with a new eligible man.*

Both segments contained one of the highest levels of withdrawal ruptures during the session, while Gloria’s dominance was relatively high, and Ellis’ dominance was relatively low compared to their typical behaviors in this session. The withdrawal ruptures were expressed through self-deprecating statements in both segments, such as Gloria describing herself as “not much of a reader” and attributing her dating struggles to internal reasons such as she being “too shy,” “defensive” or “afraid.” Thus, her dominance was not so much an effort to control or assert power over the other, but in control over the conversation via a relatively higher proportion of talking (note: Ellis talked much more frequently than Gloria throughout the session). This is an example of how the interpersonal measures reflect behavior, sometimes divorced from its underlying meaning or motivation. In other words, in this session, variation in dominance and submission primarily reflected speaking turns. Withdrawal ruptures were evident in Gloria’s somewhat deferential behaviors such as complimenting Ellis’s book and expressing hesitancy regarding her ability to find attractive partners or figure out solutions to her difficulties doing so. These moments reflected an interesting dynamic, in that Gloria was both talking about and demonstrating the problem of being talkative and active, but in a way that lacked substance – she was doing it in the session with Ellis, but also describing situations in which she did the same dynamic occurred when she was talking with men. As such, there was a risk for the same kind of rupture she described occurring in the psychotherapy session. Skillful therapists from different orientations would find value in discussing that pattern, both to reduce the risk of alliance ruptures and to help Gloria gain insight and solutions to her difficulties outside of psychotherapy.

## Discussion

Our study was the first study to examine momentary associations between alliance ruptures and interpersonal behaviors using an evidence-based personality assessment framework on dominance/agency and communion/warmth. Using the Gloria films, we identified three distinct interpersonal patterns associated with ruptures concurrently for each session: In the session with Rogers, Gloria’s increased warmth was an interpersonal signal of increases in withdrawal ruptures; in the session with Perls, Gloria’s increased dominance and decreased warmth were signals and consequences of increased confrontation ruptures; in the session with Ellis, Ellis’ decreased dominance and Gloria’s increased dominance were signals of increased withdrawal ruptures. These results showed dyadic-specific interpersonal patterns and consequences of ruptures in each session of the Gloria’s films.

The associations identified in Perls’ session were consistent with our hypothesized relationships between confrontation ruptures and interpersonal behaviors, whereas the results in the other two sessions revealed more subtlety to these relationships. Consistent with our hypothesis, confrontation ruptures manifest in a more dominant and cold manner in Gloria. In fact, this is the only session that Gloria became quite cold toward her therapist. We also extended this hypothesis and found a carryover impact of confrontation on Gloria’s warmth but not dominance over 30 s, suggesting that the conflicts may have a continuing impact on her expressed friendliness but not her expressed dominance.

In contrast, in Rogers’ session we did not observe associations between withdrawal and submissiveness as hypothesized. Instead, the results revealed a relationship in which Gloria expressed warmth to avoid expressing frustrations directly. Her seemingly warm smiles served as withdrawal gestures to cover her frustration toward Rogers when her direct questions were repeatedly not answered. This is consistent with the notion in the rupture-repair model regarding behaviors that seemed like “moving toward each other” but in fact are “moving away” from the relationship. The challenge of expressing her authentic feelings in ruptures also resonated with their main discussions in this session, which is how to be authentic and find what Gloria really needs and wants.

We found the associations that were opposite to the hypothesized relationships in the session with Ellis: withdrawal ruptures were hypothesized to relate to decreases in Gloria’s dominance, but instead were related to increases in Gloria’s dominance and decreases in Ellis’ dominance. Here a difference between interpersonal motivation and observed behaviors may explain these results. In this session, Gloria frequently made self-deprecating statements to herself and complimentary statements to Ellis, which may signal a submissive stance in motivation (although we can never be sure about other people’s motivations). However, at the level of observable behaviors, Gloria only expressed withdrawal concerns toward therapy indirectly when she became more dominant to speak and when Ellis did not talk to give back some control back to her. This result also emphasized the importance of examining within-person fluctuations in addition to between-person relationships. It is possible that patients who were more submissive compared to other patients may generally express more withdrawal ruptures, but in within-person comparisons, the expressions of withdrawal ruptures may co-occur with more dominant behaviors. Therefore, compared to moments where Gloria was even more submissive and did not express concerns, she became relatively more dominant than her typical behaviors when withdrawal ruptures occurred.

### Implications for Examining Dynamic Rupture Processes

This study specifically captured session-specific, yet repeatedly identifiable, patterns between ruptures and interpersonal behaviors within each session. Given the nature of the Gloria films, we could not replicate these patterns in additional sessions, but future studies could identify momentary patterns within each session and examine the generalizability of results in at least three ways at the individual, dyad, and group levels. At the individual level, researchers could examine the interpersonal patterns of ruptures in a critical session, and test whether the identified patterns can inform the case conceptualization, whether this pattern repeatedly shows up in consequent sessions, and whether a change in this pattern in the long term may reflect therapeutic progress in single case analyses.

At the dyadic level, researchers can examine the extent to which the interpersonal patterns of ruptures may not be based on only patient’s characteristics but also on dyadic influences. As shown in Gloria’s films, we identified three distinct patterns for the same patient. Future studies could examine how the patient-therapist interaction may change how ruptures manifest to inform therapist’s practices and awareness on dyadic influences.

Lastly at the group level, future studies could examine what extent to which the kinds of idiographic associations between ruptures and interpersonal behaviors identified here reflect nomothetic patterns at group level. Based on the results of this study, we might expect that dominance complementarity would be a nomothetic pattern characteristic of most relationships and sessions, whereas links between certain interpersonal behaviors and ruptures may vary across relationships or sessions. Knowledge about the generalizability of such patterns can be informative regarding the momentary interpersonal manifestations, predictors, and consequences of ruptures at group and individual level. Both idiographic and nomothetic effects are important, but it is useful to distinguish those kinds of patterns characteristic of one therapy dyad from those kinds of patterns predictable in psychotherapy in general.

This study was one of the first studies to mapping dimensional interpersonal behaviors with ruptures that are distinguished using a categorical model (i.e., withdrawal and/or confrontation types). Future studies may examine whether dimensional models in personality can be used to provide dimensional descriptions of styles in ruptures and whether dimensional models or categorical models may be best in capturing alliance ruptures and examining their impacts on therapeutic outcomes.

One practical implication of this study is that interpersonal ruptures can be highly repetitive and identifiable even within a single session; therefore, paying attention to these repetitive cues can enhance rupture recognition. Another clinical implication is that the between-person interpersonal patterns of ruptures may (e.g., in the session with Perls) or may not (e.g., in the session with Ellis or Rogers) signal the development of ruptures within each dyad. Therefore, we cannot only rely on between-person knowledge to identify ruptures (e.g., ignoring the possibility that patient’s increased dominance may be signals for withdrawal ruptures). Learning dyad-specific patterns of rupture manifestation is critical for clinical practice.

### Implications Regarding Connecting the AMPD More Directly to Psychotherapy

In this study we used the interpersonal circumplex model, which has explicit connections to AMPD personality assessment (Waugh et al., 2017), to conceptualize interpersonal dynamics as they unfold during psychotherapy sessions, and to relate to those dynamics to therapeutically important rupture patterns. As such, it provides a model for how to connect personality assessment and diagnosis to experience-near clinical intervention. While this proof-of-concept is preliminary, it provides both a specific paradigm and a more general nudge to close the gap between research and practice, and between diagnosis and psychotherapy. Our view is that closing the gap in this way should be a critical feature of the next generation of clinical research.

The next step with regards to the AMPD is to extend these findings to include other aspects of personality diagnosis that are not well-represented within interpersonal theory. We see two general directions for future studies along these lines, which correspond to the two AMPD criteria. First, Criterion B includes several traits related to negative affect, disinhibition, and psychoticism that vary dynamically but are not directly measured within the interpersonal circumplex framework. Future studies should examine the degree to which momentary variation on these dimensions is related to psychotherapy concepts such as therapy ruptures and other alliance-related processes. Second, Criterion A does not refer directly to the kinds of processes articulated in interpersonal theory (e.g., complementarity), but it does refer to a number of other important functional dynamics (e.g., those related to self-regulation). The interpersonal concept of complementarity provides an exemplar of how dynamic, functional concepts can be operationalized and measured. But ultimately, fulfilling the clinical potential of the AMPD and distinguishing its two primary criteria will require a more direct operationalization of the kinds of processes that are currently alluded to, but not directly measured, by Criterion A.

### Limitations

It is important to acknowledge several limitations in interpreting these results. Overall, we regard this as a proof-of-concept demonstration, not as a study from which conclusions about how to practice psychotherapy can be drawn. The Gloria’s films were produced in a special context of filming that is different from typical psychotherapy sessions. This demonstration was also conducted in a previous era and did not include psychotherapy modalities that are currently more widely practiced. Future research should extend this approach to other modalities, clinical populations, and clinicians. The rupture repair was not coded for these sessions and we used the decrease of ruptures to approximate de-escalation of ruptures. Future studies may examine how effective and ineffective repair strategies are associated with different interpersonal behaviors. Because of the brief nature of each therapy, we did not obtain the optimal number of datapoints (>100 assessments per session) to conduct DSEM analyses. We used a time interval of 30 s given that it can capture meaningful changes but testing whether results would generalize to other time intervals is also needed.

### Conclusion

The current study demonstrated a novel way to examine personality dynamics and alliance ruptures using dynamic assessment and analytic methods capable of identifying within-session linkages between relational microprocesses and withdrawal and confrontation ruptures. We found distinctive interpersonal patterns associated with each of the well-known Gloria sessions with Rogers, Perls, and Ellis. These results depicted the rich and specific information these methods can provide and point to future possibilities for examining the interpersonal processes that promote more effective psychotherapy.

## Data Availability Statement

The datasets generated for this study can be found in online repositories. The names of the repository/repositories and accession number(s) can be found below: https://osf.io/k6dh8.

## Author Contributions

XL and AL contributed to the design of the work. XL, EG, JT, KT, CH, and AL contributed to the acquisition of the data of the work. XL contributed to the analysis of the work. XL, CH, EG, JT, and AL contributed to the interpretation of the data and drafting of the work. All authors contributed to the article and approved the submitted version.

## Conflict of Interest

The authors declare that the research was conducted in the absence of any commercial or financial relationships that could be construed as a potential conflict of interest.

## Publisher’s Note

All claims expressed in this article are solely those of the authors and do not necessarily represent those of their affiliated organizations, or those of the publisher, the editors and the reviewers. Any product that may be evaluated in this article, or claim that may be made by its manufacturer, is not guaranteed or endorsed by the publisher.

## References

[B1] Altenstein D. Krieger T. Holtforth M. G. (2013). Interpersonal microprocesses predict cognitive-emotional processing and the therapeutic alliance in psychotherapy for depression. *J. Counsel. Psychol.* 60 445–452. 10.1037/a0032800 23647390

[B2] Asparouhov T. Hamaker E. L. Muthén B. (2018). Dynamic Structural Equation Models. *Struct. Equat. Model. Multidiscipl. J.* 25 359–388. 10.1080/10705511.2017.1406803

[B3] Bach B. Bernstein D. P. (2019). Schema therapy conceptualization of personality functioning and traits in ICD-11 and DSM-5. *Curr. Opin. Psychiat.* 32 38–49. 10.1097/YCO.0000000000000464 30299307

[B4] Barbosa E. Cunha C. Santos A. Gonçalves M. M. Salgado J. (2017). Narrative change in Gloria Films: Comparing various processes of therapeutic innovation. *Psychother. Res.* 27 89–101. 10.1080/10503307.2015.1072286 26308677

[B5] Boritz T. Barnhart R. Eubanks C. F. McMain S. (2018). Alliance Rupture and Resolution in Dialectical Behavior Therapy for Borderline Personality Disorder. *J. Personal. Disord.* 32 115–128. 10.1521/pedi.2018.32.supp.115 29388901

[B6] Carson R. C. (1969). *Interaction Concepts of Personality.* Chicago: Aldine, 1969.

[B7] Chen R. Atzil-Slonim D. Bar-Kalifa E. Hasson-Ohayon I. Refaeli E. (2018). Therapists’ recognition of alliance ruptures as a moderator of change in alliance and symptoms. *Psychother. Res.* 28 560–570. 10.1080/10503307.2016.1227104 27602795

[B8] Cicchetti D. V. (1994). Guidelines, criteria, and rules of thumb for evaluating normed and standardized assessment instruments in psychology. *Psychol. Assess.* 6 284–290. 10.1037/1040-3590.6.4.284

[B9] Coutinho J. Ribeiro E. Sousa I. Safran J. D. (2014). Comparing two methods of identifying alliance rupture events. *Psychotherapy* 51 434–442. 10.1037/a0032171 23668940

[B10] Dawood S. Dowgwillo E. A. Wu L. Z. Pincus A. L. (2018). “Contemporary integrative interpersonal theory of personality,” in *The SAGE Handbook of Personality and Individual Differences: The Science of Personality and Individual Differences*, eds Zeigler-Hill V. Shackelford T. K. (Newbury Park: SAGE Publications Ltd), 171–202. 10.4135/9781526451163.n8

[B11] Dowgwillo E. A. Pincus A. L. Newman M. G. Wilson S. J. Molenaar P. C. Levy K. N. (2019). “Two methods for operationalizing the interpersonal situation to investigate personality pathology and interpersonal perception in daily life,” in *Personality disorders: What we know and future directions for research*, eds Truslow L. I. Rahmaan J. M. (New York, NY: Nova Science Publishers), 31–106.

[B12] Dowgwillo E. A. Roche M. J. Pincus A. L. (2018). Examining the Interpersonal Nature of Criterion A of the DSM-5 Section III Alternative Model for Personality Disorders Using Bootstrapped Confidence Intervals for the Interpersonal Circumplex. *J. Personal. Assess.* 100 581–592. 10.1080/00223891.2018.1464016 29723070

[B13] Ellis A. (1986). Comments on Gloria. *Psychother. Theory Res. Pract. Training* 23 647–648. 10.1037/h0085671

[B14] Eubanks C. F. Hunter E. B. (2020). HiTOP and psychotherapy integration: Promising potential. *J. Psychother. Integrat.* 30 498–505. 10.1037/int0000254

[B15] Eubanks C. F. Lubitz J. Muran J. C. Safran J. D. (2019). Rupture Resolution Rating System (3RS): Development and validation. *Psychother. Res.* 29 306–319. 10.1080/10503307.2018.1552034 30526383 PMC6408286

[B16] Eubanks C. F. Muran J. C. Safran J. D. (2015). *Rupture resolution rating system (3RS): Manual.* New York, NY: Mount Sinai-Beth Israel Medical Center.

[B17] Eubanks C. F. Muran J. C. Safran J. D. (2018). Alliance rupture repair: A meta-analysis. *Psychotherapy* 55 508–519. 10.1037/pst0000185 30335462

[B18] Fournier M. A. Moskowitz D. S. Zuroff D. C. (2008). Integrating dispositions, signatures, and the interpersonal domain. *J. Personal. Soc. Psychol.* 94 531–545. 10.1037/0022-3514.94.3.531 18284296

[B19] Fox S. E. Thomas K. M. Durbin C. E. Hopwood C. J. (2021). A variance decomposition of the Continuous Assessment of Interpersonal Dynamics. *Psychol. Assess.* 33 427–442. 10.1037/pas0000995 33793264

[B20] Ghosh S. K. Mukhopadhyay P. Lu J. (2006). Bayesian analysis of zero-inflated regression models. *J. Statist. Planning Infere.* 136 1360–1375. 10.1016/j.jspi.2004.10.008

[B21] Girard J. M. Wright A. G. (2018). DARMA: Software for dual axis rating and media annotation. *Behav. Res. Methods* 50 902–909. 10.3758/s13428-017-0915-5 28634724 PMC5738301

[B22] Greenberg L. (1997). Gestalt therapy: A rough diamond. *PsycCRITIQUES* 42 196–198. 10.1037/000493

[B23] Hallgren K. A. (2012). Computing Inter-Rater Reliability for Observational Data: An Overview and Tutorial. *Tutor Quant Methods Psychol.* 8 23–34. 10.20982/tqmp.08.1.p023 22833776 PMC3402032

[B24] Hopwood C. J. (2018). A framework for treating DSM-5 alternative model for personality disorder features. *Personal. Mental Health* 12 107–125. 10.1002/pmh.1414 29656545

[B25] Hopwood C. J. Bagby R. M. Gralnick T. Ro E. Ruggero C. Mullins-Sweatt S. (2020a). Integrating psychotherapy with the hierarchical taxonomy of psychopathology (HiTOP). *J. Psychother. Integrat.* 30 477–497. 10.1037/int0000156

[B26] Hopwood C. J. Harrison A. L. Amole M. Girard J. M. Wright A. G. C. Thomas K. M. (2020b). Properties of the Continuous Assessment of Interpersonal Dynamics Across Sex. *Level Familiar. Interpers. Conflict. Assess.* 27 40–56. 10.1177/1073191118798916 30221975 PMC8898566

[B27] Hopwood C. J. Pincus A. L. Wright A. G. (2019). The interpersonal situation: Integrating personality assessment, case formulation, and intervention. *Using Basic Personal. Res. Inform Personal. Pathol.* 2019 94–121. 10.1093/med-psych/9780190227074.003.0005

[B28] Hopwood C. J. Zimmermann J. Pincus A. L. Krueger R. F. (2015). Connecting Personality Structure and Dynamics: Towards a More Evidence-Based and Clinically Useful Diagnostic Scheme. *J. Personal. Disord.* 29 431–448. 10.1521/pedi.2015.29.4.431 26200845

[B29] Horowitz L. M. (2004). *Interpersonal Foundations of Psychopathology.* Washington, DC: American Psychological Association.

[B30] Kiesler D. J. Goldston C. S. (1988). Client-therapist complementarity: An analysis of the Gloria films. *J. Counsel. Psychol.* 35 127–133. 10.1037/0022-0167.35.2.127

[B31] Lambert M. (2007). Presidential address: What we have learned from a decade of research aimed at improving psychotherapy outcome in routine care. *Psychother. Res.* 17 1–14. 10.1080/10503300601032506

[B32] Leary T. (1957). *Interpersonal Diagnosis of Personality; a Functional Theory and Methodology for Personality Evaluation.* Oxford: Ronald Press.

[B33] Li X. Kivlighan D. M. Jr. Hill C. E. (2020). Follow you or follow me? Examining therapist responsiveness to client and responsiveness to self, using differential equations model and multilevel data disaggregation from an interpersonal theory framework. *J. Counsel. Psychol.* 67 608–621. 10.1037/cou0000406 31855025

[B34] Lizdek I. Sadler P. Woody E. Ethier N. Malet G. (2012). Capturing the Stream of Behavior: A Computer-Joystick Method for Coding Interpersonal Behavior Continuously Over Time. *Soc. Sci. Comput. Rev.* 30 513–521. 10.1177/0894439312436487

[B35] Locke K. D. (2010). “Circumplex Measures of Interpersonal Constructs,” in *Handbook of Interpersonal Psychology*, eds Strack S. Horowitz L. M. (New Jersey, NJ: John Wiley & Sons, Ltd), 313–324. 10.1002/9781118001868.ch19

[B36] Morey L. C. Good E. W. Hopwood C. J. (2020). Global personality dysfunction and the relationship of pathological and normal trait domains in the DSM−5 alternative model for personality disorders. *J. Personal.* [Preprint]. 10.1111/jopy.12560 32422689

[B37] Mulay A. L. Cain N. M. Waugh M. H. Hopwood C. J. Adler J. M. Garcia D. J. (2018). Personality Constructs and Paradigms in the Alternative DSM-5 Model of Personality Disorder. *J. Personal. Assess.* 100 593–602. 10.1080/00223891.2018.1477787 29902081

[B38] Mullins-Sweatt S. N. Hopwood C. J. Chmielewski M. Meyer N. A. Min J. Helle A. C. (2020). Treatment of personality pathology through the lens of the hierarchical taxonomy of psychopathology: Developing a research agenda. *Personal. Mental Health* 14 123–141. 10.1002/pmh.1464 31364820 PMC7053295

[B39] Muran J. C. Eubanks C. F. (2020). *Therapist Performance Under Pressure: Negotiating Emotion, Difference, and Rupture.* Washington, DC: American Psychological Association.

[B40] Muran J. C. Safran J. D. Gorman B. S. Samstag L. W. Eubanks-Carter C. Winston A. (2009). The relationship of early alliance ruptures and their resolution to process and outcome in three time-limited psychotherapies for personality disorders. *Psychother. Theory Res. Pract. Training* 46 233–248. 10.1037/a0016085 22122620

[B41] Muthén L. K. Muthén B. O. (1998–2017). *Mplus 8.0.* Los Angeles, CA: Muthén & Muthén.

[B42] Pincus A. L. (2005). “A Contemporary Integrative Interpersonal Theory of Personality Disorders,” in *Major Theories of Personality Disorder*, 2nd Edn, eds Lenzenweger M. F. Clarkin J. F. (New York, NY: Guilford Press), 282–331.

[B43] Pincus A. L. (2018). An interpersonal perspective on Criterion A of the DSM-5 Alternative Model for Personality Disorders. *Curr. Opin. Psychol.* 21 11–17. 10.1016/j.copsyc.2017.08.035 28926742

[B44] Pincus A. Roche M. (2019). *Paradigms of Personality Assessment and Level of Personality Functioning in Criterion A of the AMPD.* Milton Park: Taylor and Francis, 48–59.

[B45] Ringwald W. R. Hopwood C. J. Pilkonis P. A. Wright A. G. C. (2020). Dynamic features of affect and interpersonal behavior in relation to general and specific personality pathology. *Personal. Disord. Theory Res. Treat.* 2020:er0000469. 10.1037/per0000469 33211527

[B46] Rogers C. R. (1946). Significant aspects of client-centered therapy. *Am. Psychol.* 1 415–422. 10.1037/h0060866 20280375

[B47] Rogers C. R. (1957). The necessary and sufficient conditions of therapeutic personality change. *J. Consult. Psychol.* 21 95–103. 10.1037/h0045357 13416422

[B48] Ruggero C. J. Kotov R. Hopwood C. J. First M. Clark L. A. Skodol A. E. (2019). Integrating the Hierarchical Taxonomy of Psychopathology (HiTOP) into clinical practice. *J. Consult. Clin. Psychol.* 87 1069–1084. 10.1037/ccp0000452 31724426 PMC6859953

[B49] Sadikaj G. Moskowitz D. S. Russell J. J. Zuroff D. C. Paris J. (2013). Quarrelsome behavior in borderline personality disorder: Influence of behavioral and affective reactivity to perceptions of others. *J. Abnorm. Psychol.* 122 195–207. 10.1037/a0030871 23231460

[B50] Sadler P. Ethier N. Gunn G. R. Duong D. Woody E. (2009). Are we on the same wavelength? Interpersonal complementarity as shared cyclical patterns during interactions. *J. Personal. Soc. Psychol.* 97 1005–1020. 10.1037/a0016232 19968416

[B51] Sadler P. Woody E. McDonald K. Lizdek I. Little J. (2015). A lot can happen in a few minutes: Examining dynamic patterns within an interaction to illuminate the interpersonal nature of personality disorders. *J. Personal. Disord.* 29 526–546. 10.1521/pedi.2015.29.4.526 26200850

[B52] Safran J. D. (1993). The therapeutic alliance rupture as a transtheoretical phenomenon: Definitional and conceptual issues. *J. Psychother. Integrat.* 3 33–49. 10.1037/h0101190

[B53] Safran J. D. Muran J. C. (2006). Has the concept of the therapeutic alliance outlived its usefulness? *Psychother. Theory Res. Pract. Training* 43 286–291. 10.1037/0033-3204.43.3.286 22122099

[B54] Sharf J. Primavera L. H. Diener M. J. (2010). Dropout and therapeutic alliance: A meta-analysis of adult individual psychotherapy. *Psychother. Theory Res. Pract. Training* 47 637–645. 10.1037/a0021175 21198249

[B55] Shostrom E. L. (1966). *Three approaches to psychotherapy [Film].* Santa Ana, CA: Psychological Films.

[B56] Sleep C. E. Weiss B. Lynam D. R. Miller J. D. (2020). The DSM–5 section III personality disorder criterion a in relation to both pathological and general personality traits. *Personal. Disord. Theory Res. Treat.* 11 202–212. 10.1037/per0000383 31804130

[B57] Sullivan H. S. (1953). *The Interpersonal Theory of Psychiatry.* New York, NY: W W Norton & Co.

[B58] Thomas K. M. Hopwood C. J. Woody E. Ethier N. Sadler P. (2014). Momentary assessment of interpersonal process in psychotherapy. *J. Counsel. Psychol.* 61 1–14. 10.1037/a0034277 24001123

[B59] Wang X. Chen M. Kuo R. C. Dey D. K. (2015). Bayesian spatial-temporal modeling of ecological zero-inflated count data. *Statist. Sin.* 25 189–204. 10.5705/ss.2013.212w 26997848 PMC4793368

[B60] Waugh M. H. Hopwood C. J. Krueger R. F. Morey L. C. Pincus A. L. Wright A. G. C. (2017). Psychological assessment with the DSM–5 Alternative Model for Personality Disorders: Tradition and innovation. *Profess. Psychol. Res. Pract.* 48 79–89. 10.1037/pro0000071 28450760 PMC5403154

[B61] Wiggins J. S. (1979). A psychological taxonomy of trait-descriptive terms: The interpersonal domain. *J. Personal. Soc. Psychol.* 37 395–412. 10.1037/0022-3514.37.3.395

[B62] Wiggins J. S. (1991). “Agency and communion as conceptual coordinates for the understanding and measurement of interpersonal behavior,” in *Thinking Clearly About Psychology: Essays in Honor of Paul E. Meehl, Vol. 1, Matters of Public Interest; Vol. 2. Personality and Psychopathology*, eds Cicchetti D. Grove W. M. (Minneapolis: University of Minnesota Press), 89–113.

[B63] Wilson S. Stroud C. B. Durbin C. E. (2017). Interpersonal dysfunction in personality disorders: A meta-analytic review. *Psychol. Bull.* 143:677. 10.1037/bul0000101 28447827 PMC5507693

[B64] Wright A. G. C. Pincus A. L. Hopwood C. J. Thomas K. M. Markon K. E. Krueger R. F. (2012). An Interpersonal Analysis of Pathological Personality Traits in DSM-5. *Assessment* 19 263–275. 10.1177/1073191112446657 22589411 PMC3405202

[B65] Wright A. G. C. Pincus A. Hopwood C. (2020). Contemporary Integrative Interpersonal Theory: Integrating Structure, Dynamics, Temporal Scale, and Levels of Analysis. 10.31234/osf.io/fknc8 [Preprint]. 37126059

[B66] Wright A. G. Kaurin A. (2020). Integrating structure and function in conceptualizing and assessing pathological traits. *Psychopathology* 53 189–197. 10.1159/000507590 32375147

[B67] Yang M. Cavanaugh J. E. Zamba G. K. (2015). State-space models for count time series with excess zeros. *Statist. Modell.* 15 70–90. 10.1177/1471082X14535530

