# Corrigendum: Using interpersonal dimensions of personality and personality pathology to examine momentary and idiographic patterns of alliance rupture

**DOI:** 10.3389/fpsyg.2022.980807

**Published:** 2022-09-08

**Authors:** Xiaochen Luo, Christopher J. Hopwood, Evan W. Good, Joshua E. Turchan, Katherine M. Thomas, Alytia A. Levendosky

**Affiliations:** ^1^Department of Counseling Psychology, Santa Clara University, Santa Clara, CA, United States; ^2^Department of Psychology, University of Zurich, Zurich, Switzerland; ^3^Department of Psychology, Michigan State University, East Lansing, MI, United States; ^4^Counseling & Psychiatric Services, Michigan State University, East Lansing, MI, United States; ^5^Center for Therapeutic Assessment, Austin, TX, United States

**Keywords:** Alternative Model of Personality Disorders (AMPD), interpersonal circumplex model, alliance rupture, momentary processes, idiographic analysis, psychotherapy process

This is the corrigendum to the original article “*Using Interpersonal Dimensions of Personality and Personality Pathology to Examine Momentary and Idiographic Patterns of Alliance Rupture*” (Luo et al., 2021). The original article examined momentary patterns of interpersonal behaviors and alliance ruptures in each of the psychotherapy demonstration videos between the patient Gloria and three prominent therapists. In the original article, we depicted the diagrams of the dynamic structural equation modeling for each session in Figures 2, 4, 6. In these diagrams, we showed two types of effects (1) an autoregression effect for each variable, in which one variable at time *t* predicts its state at time *t* + *1* (1 refers to the unit of time interval in the model); and (2) the concurrent associations among interpersonal variables and among rupture variables at time *t* + 1. The original figures of Figures 2, 4, 6 wrongly depicted the concurrent associations for variables at time *t* instead of variables at time *t*+1. The correct versions of the Figures 2, 4, 6 are updated and included below.

**Figure 2:**
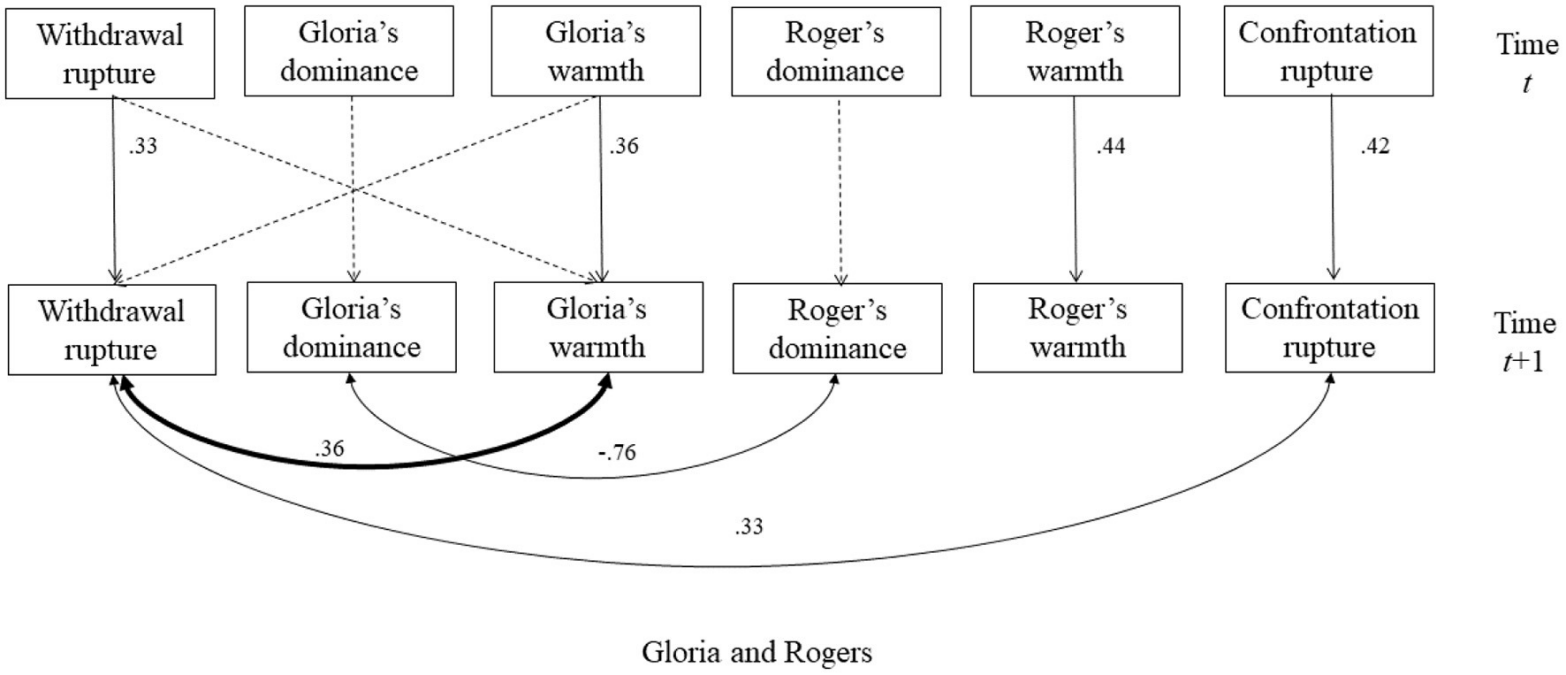
The selected model for the session with Rogers. The dash lines indicated non-significant autoregressive or cross-lagged regressive pathways. Significant parameters were noted next to solid lines. The double-arrowed lines indicated covariance and the single-arrowed lines indicated autoregressive or cross-lagged regressive pathways. The significant relationships between interpersonal behaviors and ruptures were bolded.

**Figure 4:**
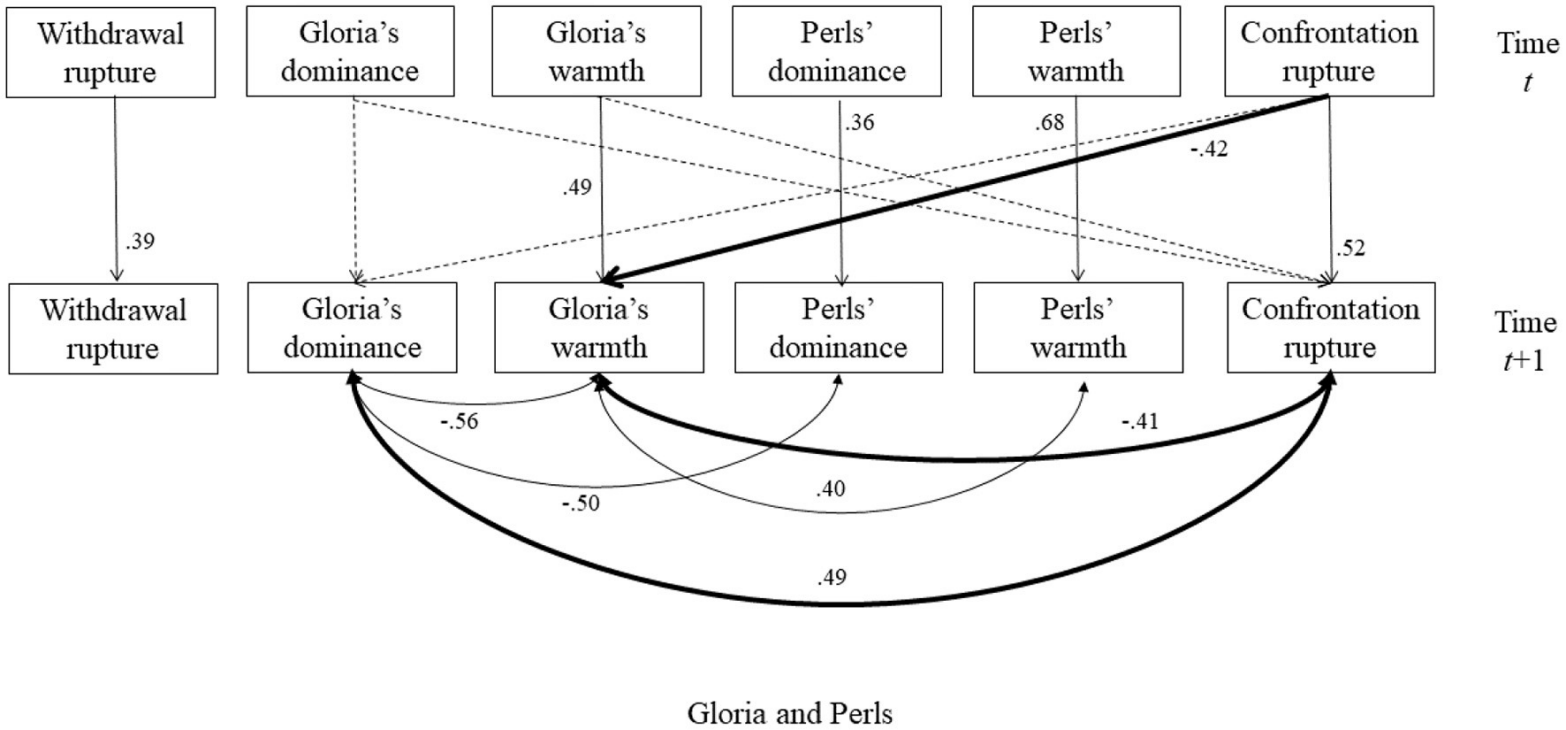
The selected model for the session with Perls. The dash lines indicated non-significant autoregressive or cross-lagged regressive pathways. Significant parameters were noted next to solid lines. The double-arrowed lines indicated covariance and the single-arrowed lines indicated autoregressive or cross-lagged regressive pathways. The significant relationships between interpersonal behaviors and ruptures were bolded.

**Figure 6:**
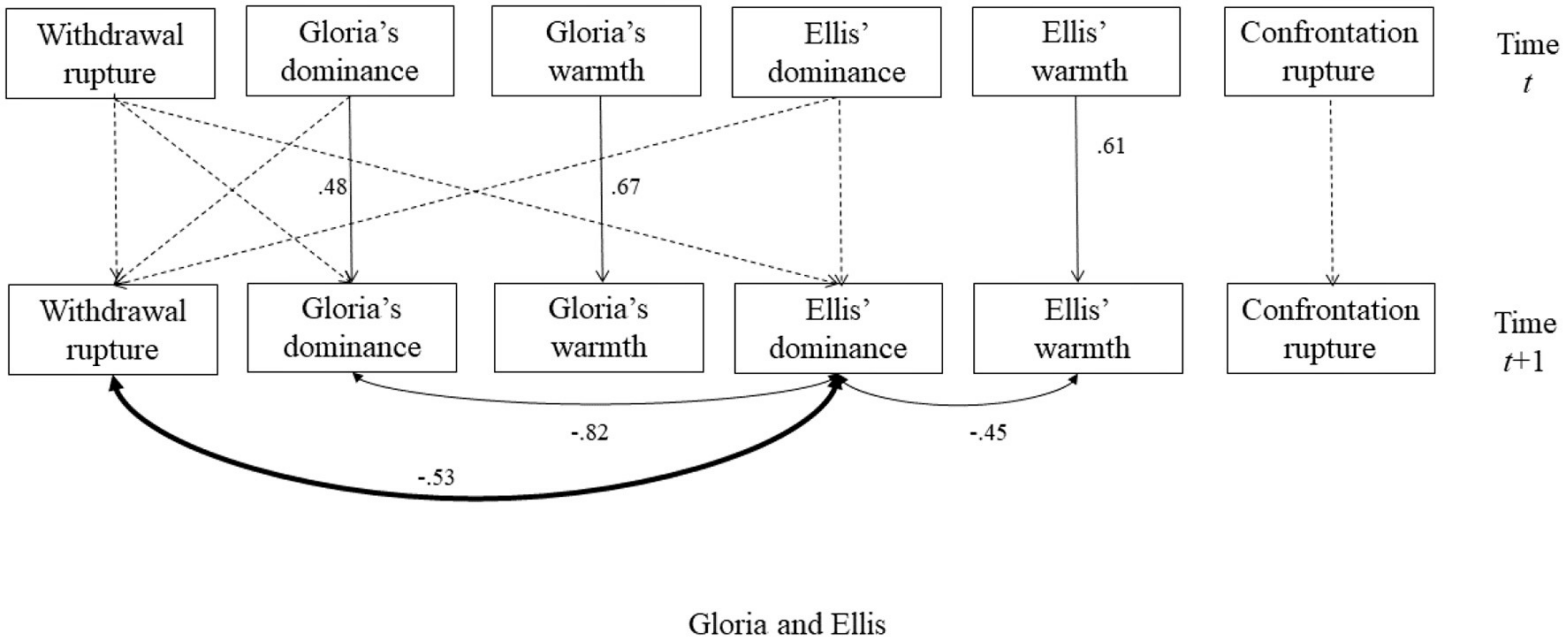
The selected model for the session with Ellis. The dash lines indicated non-significant autoregressive or cross-lagged regressive pathways. Significant parameters were noted next to solid lines. The double-arrowed lines indicated covariance and the single-arrowed lines indicated autoregressive or cross-lagged regressive pathways. The significant relationships between interpersonal behaviors and ruptures were bolded. The association between Gloria's dominance and withdrawal ruptures became non-significant (*p* = 0.047, 95% CI is −0.06 to 0.69) after adding the cross-lagged associations.

The authors apologize for this error and state that this does not change the scientific conclusions of the article in any way. The original article has been updated.

## Publisher's note

All claims expressed in this article are solely those of the authors and do not necessarily represent those of their affiliated organizations, or those of the publisher, the editors and the reviewers. Any product that may be evaluated in this article, or claim that may be made by its manufacturer, is not guaranteed or endorsed by the publisher.
